# Psittacid alphaherpesvirus 5 infection in Indian ringneck parakeets in southern California

**DOI:** 10.1177/10406387221136568

**Published:** 2022-11-10

**Authors:** Eileen E. Henderson, Nicolas Streitenberger, Javier Asin, Anibal Armien, Beate M. Crossley, April L. Childress, James F. X. Wellehan, Francisco A. Uzal

**Affiliations:** California Animal Health and Food Safety Laboratory System, University of California–Davis, San Bernardino branches, CA, USA; California Animal Health and Food Safety Laboratory System, University of California–Davis, San Bernardino branches, CA, USA; California Animal Health and Food Safety Laboratory System, University of California–Davis, San Bernardino branches, CA, USA; Davis branches, CA, USA; Davis branches, CA, USA; Department of Comparative, Diagnostic, and Population Medicine, College of Veterinary Medicine, University of Florida, Gainesville, FL, USA; Department of Comparative, Diagnostic, and Population Medicine, College of Veterinary Medicine, University of Florida, Gainesville, FL, USA; California Animal Health and Food Safety Laboratory System, University of California–Davis, San Bernardino branches, CA, USA

**Keywords:** avian, herpesvirus, Indian ringneck parakeet, *Psittacula krameria*, respiratory

## Abstract

Four Indian ringneck parakeets (*Psittacula krameri*; syn. ringneck parrots or rose-ringed parakeets) were submitted by 2 private owners for autopsy following a history of dyspnea and death. Gross findings were varied and included thickening of the left caudal thoracic air sac, white spots throughout the liver, mild dilation of the proventriculus, coelomic effusion, splenomegaly, and pulmonary congestion and edema. Microscopically, the submitted parakeets had significant lesions in the lower respiratory tract, including necrotizing bronchitis, parabronchitis, and interstitial pneumonia with numerous syncytia containing eosinophilic intranuclear inclusions. Electron microscopy of the lungs was compatible with a herpesviral infection and Psittacid alphaherpesvirus 5 (PsAHV5) was detected via PCR and sequencing. There has been inconsistent terminology used with Psittacid alphaherpesvirus 3 and PsAHV5; we attempt here to clarify the reported history of these viruses.

*Herpesviridae* is a large family of double-stranded DNA viruses that can infect diverse hosts in clade *Amniota*, infecting reptiles, birds, and mammals.^5^ A hallmark of this family of viruses is persistent infection or latency, with periodic or continuous shedding. Herpesviruses of significance in birds include *Gallid alphaherpesvirus 1* (GaAHV1; causative agent of infectious laryngotracheitis), *Gallid alphaherpesvirus 2* (GaAHV2; causative agent of Marek disease), *Anatid alphaherpesvirus 1* (AnAHV1; causative agent of duck viral enteritis), *Psittacid alphaherpesvirus 1* (PsAHV1; causative agent of papillomatosis and Pacheco disease), and *Columbid alphaherpesvirus 1* (CoAHV1; fatal disease in hawks, owls, and falcons). Herpesviruses are subclassified into subfamilies *Alphaherpesvirinae*, *Betaherpesvirinae*, and *Gammaherpesvirinae*.^5^ *Alphaherpesvirinae* is further subdivided into 5 genera: *Iltovirus*, *Mardivirus*, *Scutavirus*, *Simplexvirus*, and *Varicellovirus*. Herpesviruses within the genus *Iltovirus* (GaAHV1, PsAHV1) are often associated with respiratory disease in birds.^11^

In parrots, at least 5 herpesviruses have been named. PsAHV1 has diverse genotypes associated with different hosts and likely represents more than one species; some of these are listed in GenBank under the name “psittacid herpesvirus 4”.^16^ The provisionally named Psittacid alphaherpesvirus 2 is from a captive African grey parrot (*Psittacus erithacus*) with cloacal papillomatosis.^13^ The provisionally named Cacatuid alphaherpesvirus 1 was described from Australia in a wild sulfur-crested cockatoo (*Cacatua galerita*) with significant comorbidity.^2^ The provisionally named Cacatuid alphaherpesvirus 2 was described from a wild-caught little corella (*Cacatua sanguinea*) with nonspecific clinical signs.^15^ All of these viruses cluster in the genus *Iltovirus*.

Psittacid alphaherpesvirus 3 (PsAHV3), also clustering in *Iltovirus*, was first described from captive Bourke’s parrots (*Neopsephotus bourkii*) with respiratory disease in the USA based on a region of the DNA-dependent DNA polymerase gene (GenBank JX028240).^11^ PsAHV3 was later described from captive eclectus parrots (*Eclectus roratus*) with respiratory disease in Australia,^4^ but the sequence is not available in public databases. In 2019, the full genome of a herpesvirus was sequenced from an outbreak in Australia in a flock of captive Indian ringneck parrots (*Psittacula krameri*), associated with severe lower respiratory tract disease and high morbidity and mortality in affected birds (GenBank MK955929).^14^ The virus was called Psittacid alphaherpesvirus 5 (PsAHV5); however, the sequence had 99.78% nucleotide homology and 100% predicted amino acid homology with the available Bourke’s parrot PsAHV3 sequence over 920 bp of the DNA-dependent DNA polymerase gene, which is not consistent with a different viral species. In 2020, a herpesvirus was reported from Brazil in captive Indian ringneck parrots with respiratory disease, based on a region of the DNA-dependent DNA polymerase gene (GenBank MK922358), which had only 89.5% nucleotide homology with other reported PsAHV3 and PsAHV5 sequences, consistent with a distinct species.^7^ In early 2022, a herpesvirus was reported from Europe in captive Indian ringneck parrots and Alexandrine parakeets (*Psittacula eupatria*) with respiratory disease; partial DNA-dependent DNA polymerase sequences (GenBank OK665682–4) were identical to the viruses from the USA Bourke’s parrot PsAHV3 and Australian Indian ringneck PsAHV5 sequences.^3^ The name “Psittacid herpesvirus 3” had previously also been used in GenBank not only with the Brazilian Indian ringneck parrot virus but also what was reported as PsAHV1 genotype 3. The genetic distances among the PsAHV1 genotypes are more consistent with interspecies differences than intraspecies differences, suggesting that PsAHV1 may be divided in the future. To avoid future confusion over the name PsAHV3, and because of the consistent sequence available under the name PsAHV5, we hereafter refer to this virus as PsAHV5.

In late August and early September 2021, 4 Indian ringneck parakeets with a history of dyspnea before death were submitted by 2 private owners to the San Bernardino branch of the California Animal Health and Food Safety Laboratory (CAHFS; University of California–Davis) for autopsy and diagnostic work-up. All 4 parakeets were in good nutritional condition, and postmortem decomposition ranged from mild to advanced. Gross findings were varied and included thickening of the left caudal thoracic air sac (parakeet 1), ~2-mm diameter white spots throughout the liver (parakeet 2), mild dilation of the proventriculus (parakeet 2), coelomic effusion (parakeet 3), splenomegaly (parakeet 4), and congested and edematous lungs (parakeets 3, 4).

Samples from brain, heart, kidney, spleen, liver, lung, esophagus, trachea, proventriculus, ventriculus, small intestine, pancreas, large intestine, sciatic nerve, air sac, adrenal gland, and/or skeletal muscle were collected and fixed by immersion in 10% neutral-buffered formalin (pH 7.2) for a minimum of 24 h. These tissues were processed routinely to obtain 4-µm thick H&E-stained sections.

Three of the submitted parakeets had microscopic lesions within the lungs. These lesions included the presence of large syncytia within bronchi and parabronchi, with large amphophilic-to-eosinophilic intranuclear inclusion bodies that marginalized the chromatin, and an exudate comprised of foamy macrophages, pale basophilic mucus, homogeneously eosinophilic edema fluid, fibrin, cellular debris, and sloughed necrotic epithelial cells mostly within the parabronchi but also present in the bronchi to a lesser degree (Fig. 1). In parakeet 1, syncytia and intranuclear inclusions were also visible in the trachea, air sac(s) (Fig. 1), and infraorbital sinuses. Parakeets 1 and 3 had a concurrent fungal respiratory infection characterized by heterophilic granulomas with numerous intralesional, 4–6-µm wide, parallel-walled, septate, and dichotomous angle-branching fungal hyphae morphologically consistent with *Aspergillus* spp. within the lungs (parakeet 3) or air sacs (parakeet 1).

**Figure 1.**
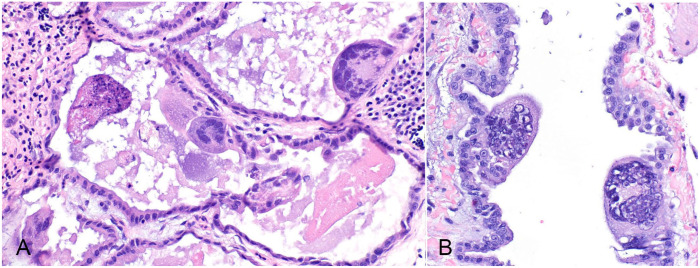
Herpesvirus-associated pneumonia in Indian ringneck parakeets. Syncytia containing amphophilic intranuclear inclusions within the **A.** lung and **B.** air sacs. H&E.

Transmission electron microscopy was subsequently performed. Briefly, formalin-fixed, 1–2-mm^3^ lung fragments were post-fixed in modified Karnovsky, 1% osmium tetroxide, and 0.1 M buffered cacodylate before being embedded in resin and sectioned for electron microscopy. At the ultrastructural level, inclusions observed on light microscopy were consistent with herpesviral replication complexes (Fig. 2). These replication complexes consisted of variably dense, amorphous-to-filamentous viroplasm containing completely and incompletely assembled 90–100-nm diameter capsids. Primary enveloped virions were present in the perinuclear space. In the cytoplasm, nucleocapsids acquired tegument proteins as electron-dense deposits and formed mature virions inside vesicles (secondary envelopment).

**Figure 2.**
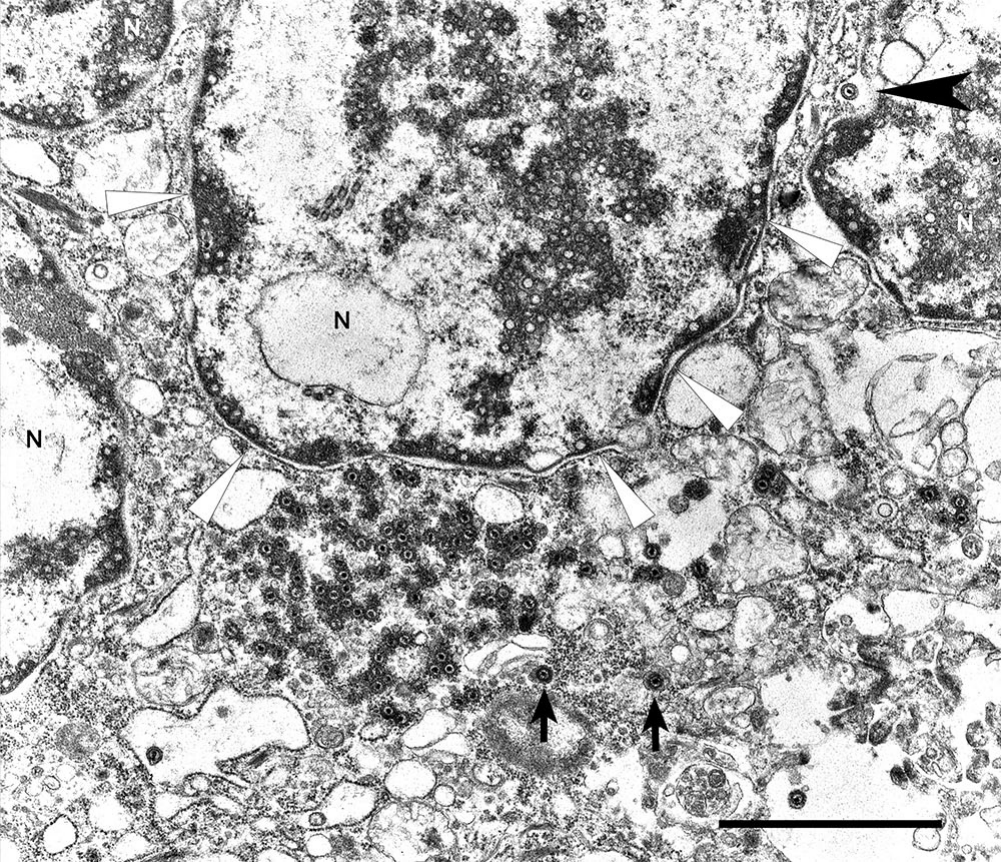
Syncytial cell with nuclear replication and assembly and cytoplasmic replication complexes. Note numerous nucleocapsids positioned at the nuclear membrane. A maturing enveloped virion is trafficking through the nuclear membrane (black arrowhead). Virions near cisterns (arrows) are gaining the final envelope and tegmental proteins. N = nuclei. The white arrowheads indicate the nuclear margin of one nucleus. Bar = 2 µm.

For herpesviral testing, DNA was extracted from the samples (DNeasy kit; Qiagen). Nested PCR amplification of a partial sequence of the herpesviral DNA-dependent DNA polymerase gene was also performed using methods described previously.^18^ The product was resolved on 1% agarose gel and purified (QIAquick gel extraction kit; Qiagen). Direct sequencing was performed (Big-Dye terminator kit; Applied Biosystems). Primer sequences were edited out prior to further analysis. All 4 samples produced resulted in identical sequences of 187 bp, which were compared to those in GenBank using BLASTN and found to have 100% nucleotide homology with PsAHV5 (GenBank MK955929, OK665682–4) from Australia and Europe, and 92.5% homology with PsAHV3 (GenBank MK922358) from Brazil.^1^ There was no overlap with the PsAHV3 sequence from the USA (GenBank JX028240); the PsAHV3 sequence from the eclectus parrot in Australia is not available. A representative sequence from our cases was deposited in GenBank (OP222575).

Ancillary tests, including bacterial and fungal cultures, virus isolation (VI), PCR for several viral and bacterial agents, fecal parasitologic examination, and toxicology (heavy metal screen) were performed following CAHFS standard operating procedures (Table 1). PCR testing for avian paramyxovirus 1 (APMV1; *Avian orthoavulavirus 1*) and avian influenza A virus (*Alphainfluenzavirus influenzae*) was performed using the protocol provided by the National Animal Health Laboratory Network.^12,19^ Briefly, parakeet 1 was confirmed to be infected with *West Nile virus* (WNV) via immunohistochemistry, PCR, and VI. Additionally, a reovirus was detected by VI in parakeet 3, and *Aspergillus* spp. was cultured from the air sac of parakeet 1. All other ancillary test results were negative or non-diagnostic. Based on the lesions and results of ancillary tests, a diagnosis of pneumonia caused by PsAHV5 was established in our 4 parakeets.

**Table 1.** Results of ancillary testing performed on 4 parakeets, including cultures (aerobic, anaerobic, fungal), PCR assays (avian influenza A virus [AIV], avian paramyxovirus 1 [APMV1], psittacid herpesvirus 1 [PsHV1], West Nile virus [WNV], chlamydia, *Pasteurella multocida*), virology (virus isolation [VI]), toxicology (heavy metal screen [HMS]), and parasitology (fecal examination).

	Bacteriology			Biotechnology–PCRs						Virology	Toxicology	Parasitology
	Aerobic culture	Anaerobic culture	Fungal culture	AIV	APMV1	PsHV1	WNV	Chlamydia	*Pasteurella multocida*	VI	HMS	Fecal examination
Parakeet 1 (submission 1)	NSF	NA	*Aspergillus* spp.	−	−	−	+	−	−	WNV	NSF	−
Parakeet 2 (submission 2)	NSF	NSF	NA	−	−	−	NA	NA	NA	NSF	NSF	−
Parakeet 3 (submission 2)	NSF	NSF	NA	−	−	−	NA	NA	NA	Reovirus	NSF	−
Parakeet 4 (submission 2)	NSF	NSF	NA	−	−	−	NA	NA	NA	NSF	NSF	−

NA = not applicable or not performed; NSF = no significant findings; − = negative or not detected; + = positive.

Previous reports of PsAHV3 and PsAHV5 were associated with respiratory lesions similar to those described in our cases, including the formation of large syncytia and intranuclear inclusions. Reports of a respiratory herpesvirus in *Psittacula* date back to the 1990s,^17^ including a flock of Indian ringneck parakeets imported to the United States from Australia.^6^ In these case reports, molecular characterization of the virus was not pursued.^6,17^

To date, PsAHV5 has been documented in Indian ringneck parakeets, Alexandrine parakeets, Bourke’s parrots, and possibly eclectus parrots, although the sequence is not available. Herpesviruses are generally well-adapted to their host species, and severe disease is much less common in endemic hosts than aberrant hosts. Indian ringneck parrots and Alexandrine parakeets are both in the genus *Psittacula*, and eclectus parrots are very closely related, in tribe *Psittaculini*.^9^ However, Bourke’s parrots, in the tribe *Pezoporini*, with the ground parrots (*Pezoporus* sp.) and grass parrots (*Neophema* sp.), are not particularly closely related to *Psittaculini*, and all *Psittacidae* may potentially be at risk.^10^ Further work on the host range of PsAHV5 is needed.

PsAHV5 appears to have a particular tropism for the lower respiratory tract. Although lesions were described in the trachea, sinuses, and air sacs of one parakeet, these manifestations of the virus appear to be less common. Two of the birds had concurrent fungal respiratory tract infections. *Aspergillus* spp. are typically opportunistic pathogens, and mycotic respiratory tract infections are common, particularly in immunosuppressed individuals. The significance of the reovirus isolated from one of the parakeets is unclear. One parakeet also had histologic lesions compatible with a flaviviral infection, and WNV was detected via 3 different tests. WNV is known to produce severe systemic disease in a wide range of psittacines.^8^ Similar to birds of prey (e.g., *Strigiformes*, *Falconiformes*), microscopic lesions in affected psittacines are typically characterized by chronic lymphoplasmacytic and histiocytic inflammation of the heart, spleen, pancreas, liver, heart, and/or intestine. Co-infections are not reported commonly.

In psittacids with a history of respiratory disease, herpesviral infection should be considered as a differential diagnosis. Unfortunately, histology alone is insufficient to diagnose PsAHV5 infection given that other related herpesviruses (e.g., the Brazilian virus described as PsAHV3) can produce similar microscopic lesions. The etiology must be confirmed by VI and/or molecular techniques. Our cases also suggest that concurrent infections with other agents (e.g., bacterial, viral, fungal) can occur.
